# Exploring the Contribution to ADHD of Genes Involved in Mendelian Disorders Presenting with Hyperactivity and/or Inattention

**DOI:** 10.3390/genes13010093

**Published:** 2021-12-30

**Authors:** Noèlia Fernàndez-Castillo, Judit Cabana-Domínguez, Djenifer B. Kappel, Bàrbara Torrico, Heike Weber, Klaus-Peter Lesch, Oscar Lao, Andreas Reif, Bru Cormand

**Affiliations:** 1 Departament de Genètica, Microbiologia i Estadística, Facultat de Biologia, Universitat de Barcelona, 08028 Barcelona, Spain; barticoa@gmail.com (B.T.); bcormand@ub.edu (B.C.); 2 Centro de Investigación Biomédica en Red de Enfermedades Raras (CIBERER), Instituto de Salud Carlos III, 28029 Madrid, Spain; 3 Institut de Biomedicina de la Universitat de Barcelona (IBUB), 08028 Barcelona, Spain; 4 Institut de Recerca Sant Joan de Déu (IR-SJD), 08950 Esplugues de Llobregat, Spain; 5 Psychiatric Genetics Unit, Group of Psychiatry, Mental Health and Addiction, Vall d’Hebron Research Institute (VHIR), Universitat Autònoma de Barcelona, 08035 Barcelona, Spain; 6 Department of Psychiatry, Hospital Universitari Vall d’Hebron, 08035 Barcelona, Spain; 7 Biomedical Network Research Centre on Mental Health (CIBERSAM), 28029 Madrid, Spain; 8 Division of Psychological Medicine and Clinical Neurosciences, MRC Centre for Neuropsychiatric Genetics and Genomics, School of Medicine, Cardiff University, Cardiff CF10 3AT, UK; djenifer.kappel@hotmail.com; 9 Department of Psychiatry, Psychosomatic Medicine and Psychotherapy, University Hospital Frankfurt, 60590 Frankfurt, Germany; weber_h2@ukw.de (H.W.); andreas.reif@kgu.de (A.R.); 10 Department of Psychiatry, Psychosomatic Medicine and Psychotherapy, University Hospital Würzburg, 97080 Wurzburg, Germany; 11 Division of Molecular Psychiatry, Center of Mental Health, University of Würzburg, 97080 Wurzburg, Germany; kplesch@mail.uni-wuerzburg.de; 12 Department of Psychiatry and Neuropsychology, School for Mental Health and Neuroscience, Maastricht University, 6221 LK Maastricht, The Netherlands; 13 Laboratory of Psychiatric Neurobiology, Institute of Molecular Medicine, I.M Sechenov First Moscow State Medical University, 119435 Moscow, Russia; 14 CNAG-CRG, Centre for Genomic Regulation (CRG), 08028 Barcelona, Spain; oscar.lao@cnag.crg.eu; 15 Barcelona Institute of Science and Technology (BIST), 08036 Barcelona, Spain; 16 Universitat Pompeu Fabra (UPF), 08002 Barcelona, Spain

**Keywords:** ADHD, rare mendelian disorders, genetic variants

## Abstract

Attention-deficit hyperactivity disorder (ADHD) is a complex neurodevelopmental disorder characterized by hyperactivity, impulsivity, and/or inattention, which are symptoms also observed in many rare genetic disorders. We searched for genes involved in Mendelian disorders presenting with ADHD symptoms in the Online Mendelian Inheritance in Man (OMIM) database, to curate a list of new candidate risk genes for ADHD. We explored the enrichment of functions and pathways in this gene list, and tested whether rare or common variants in these genes are associated with ADHD or with its comorbidities. We identified 139 genes, causal for 137 rare disorders, mainly related to neurodevelopmental and brain function. Most of these Mendelian disorders also present with other psychiatric traits that are often comorbid with ADHD. Using whole exome sequencing (WES) data from 668 ADHD cases, we found rare variants associated with the dimension of the severity of inattention symptoms in three genes: *KIF11*, *WAC*, and *CRBN*. Then, we focused on common variants and identified six genes associated with ADHD (in 19,099 cases and 34,194 controls): *MANBA*, *UQCC2*, *HIVEP2*, *FOPX1*, *KANSL1*, and *AUH*. Furthermore, *HIVEP2*, *FOXP1*, and *KANSL1* were nominally associated with autism spectrum disorder (ASD) (18,382 cases and 27,969 controls), as well as *HIVEP2* with anxiety (7016 cases and 14,475 controls), and *FOXP1* with aggression (18,988 individuals), which is in line with the symptomatology of the rare disorders they are responsible for. In conclusion, inspecting Mendelian disorders and the genes responsible for them constitutes a valuable approach for identifying new risk genes and the mechanisms of complex disorders.

## 1. Introduction

Attention-deficit hyperactivity disorder (ADHD) is characterized by symptoms of hyperactivity, increased impulsivity, and/or inattention, affecting approximately 5% of children and adolescents and 2.5% of adults worldwide [1]. There is a high comorbidity between ADHD and other psychiatric conditions such as autism spectrum disorder (ASD, 65–80%), oppositional defiant disorder (ODD, 50–60%), and conduct disorder (CD, 20–50% in children and 40–50% in adolescents), the last two being characterized by aggressive behavior [2]. Additionally, depression (16–26%) or anxiety (10–40%) disorders and obsessive-compulsive disorders (OCD, 6–15%) are highly prevalent in ADHD, especially in adult patients [2,3].

The contribution of genetic factors to ADHD is estimated to be around 70–80% in children and adults [1]. ADHD symptoms are attributable to heritable quantitative traits distributed in a continuum in the population, and diagnoses correspond to the extreme tail of these symptoms, that cause impairment. The genetics of ADHD is complex and polygenic, which represents a challenge for identifying genes involved in this neurodevelopmental disorder. Recent advances towards identifying genetic risk variants for ADHD, both common and rare, have been possible through genome-wide association study (GWAS) meta-analyses and whole-exome sequencing studies (WES) [4,5].

Some known rare Mendelian diseases, such as Phenylketonurya, Adrenoleukodystrophy, Tuberous sclerosis, Fragile X, Muchopolysacaridosis type III, Hyperlysinemia, or Joubert syndrome, include symptoms of hyperactivity and/or inattention. These disorders also present with other psychiatric symptoms that are comorbid with ADHD, as described in the Online Mendelian Inheritance in Man (OMIM) database (https://www.omim.org/). Around 7000 rare diseases have been described, affecting 350 million people globally, and often including a wide range of symptoms. The vast majority are characterized by Mendelian inheritance, and most of them (80%) have a known genetic cause [6,7]. In the last decades, and with the appearance of high-throughput and next-generation sequencing techniques, there has been a rapid advance in identifying the causal genes responsible for Mendelian disorders, which are nowadays known for more than 6000 of OMIM entries. Given their simple pattern of inheritance, they are easier to study than complex disorders, from a genetic perspective [7]. An interesting study considered this fact and searched for ADHD risk genes in three extended pedigrees with multiple members affected by ADHD, with an apparent dominant inheritance pattern [8]. Combining linkage analysis in these families and WES in ADHD patients, they identified 12 genes associated with the disorder, which points to this approach as being potentially useful to identify novel risk genes for this disorder.

In the present study, we explored the OMIM database to search for rare disorders presenting with hyperactivity and/or inattention with the objective to elucidate molecular mechanisms involved in these traits. Then, under the hypothesis that genes responsible for these Mendelian disorders may also be involved in ADHD and in its most frequent comorbidities, we investigated the possible contribution of rare and common risk variants on them using a gene-based analysis. In addition, for the common variants located in genes associated with ADHD, we investigated their role on gene expression and on impacting subcortical brain volumes. Finally, we explored whether these genes were also significantly associated with several ADHD comorbid conditions.

## 2. Materials and Methods

### 2.1. Gene Selection

We queried the OMIM database (https://www.omim.org/; accessed on 25 February 2019) with the search term “hyperactivity OR hyperactive OR attention OR inattention”. We did not include a search for the presence of impulsivity only, since this is frequently present in many disorders related to aggression and not hyperactivity. Therefore, we included impulsivity only when it is presented with hyperactivity, to avoid the inclusion of rare disorders with symptomatology not directly related to ADHD.

This search returned 182 OMIM entries that were manually curated to discard 18 entries not related to ADHD, as well as 27 entries without a known associated gene, ending up with 137 OMIM entries linked to 139 genes. From those entries, we retrieved information on related psychiatric and neurological disorders (Supplementary Table S1). From a final list of 139 OMIM genes, enrichment analyses of biological processes (Gene Ontology, GO) and KEGG pathways were performed using the WEB-based GEne SeT AnaLysis Toolkit 2019 (WebGestalt, http://www.webgestalt.org/) [9].

### 2.2. Whole-Exome Sequencing

To assess the contribution of rare variants in the OMIM gene list, we analyzed the presence of high-impact rare variants within those genes and their associations with ADHD symptom dimensions in a sample of adult patients with ADHD. The dataset comprised 668 German adults with persistent ADHD, diagnosed according to DSM-IV criteria; the mean age was 34 (ranging from 18–65), with 50.8% of the sample being males. This sample is part of the International Multicenter persistent ADHD Collaboration (IMpACT, http://www.impactadhdgenomics.com) and is described in more detail by Rovira et al. [10]. All participants signed informed consent, and the Ethics Committees of the universities involved approved the study.

Participants provided EDTA-blood samples for DNA extraction using a standard salting-out method [11]. Genomic DNA was targeted with a BGI exome capture kit and sequenced on the Illumina HiSeq2000 platform. We selected variants with a minor allele frequency (MAF) <0.01 in the European superpopulation of 1000 genomes Phase 3 [12] and extracted the variants that were more likely to affect protein function, expression, or availability: protein-truncating variants (nonsense and splice site changes) and putative damaging missense variants, using CADD_phred scores >15.

### 2.3. Rare Variant Association Analyses

Using a gene-based approach, we analyzed the cumulative effect of high-impact rare variants in the OMIM-derived genes with ADHD symptoms dimensions (inattention and hyperactivity/impulsivity symptoms). The statistical analyses were performed using the Multi-marker Analysis of GenoMic Annotation (MAGMA v1.07) software, with a SNP-wise mean model for the gene-based association analysis, adjusting for genomic principal components as covariates. We considered the genes to be associated if they reached the Bonferroni-corrected adjusted threshold for the number of genes tested (e.g., 101 genes; *p* < 4.95 × 10^−4^) or a 10% False Discovery Rate (FDR). As WES analyses were not performed for variants in the X chromosome, only genes located in the autosomes could be analyzed.

Additionally, we investigated the possibility that the OMIM gene list carried a higher-than-expected frequency of high-impact rare variants, using a Monte-Carlo resampling and permutation approach. For this, we sampled 100,000 sets of the same number of genes as those containing high-impact rare variants and analyzed the distribution of variants in those sets.

### 2.4. Common Variant Gene-Based Association Studies

The contribution of common variants (MAF > 0.01) in the OMIM gene list to ADHD was assessed through a gene-based association analysis using MAGMA v1.06 [13] and the summary statistics from the largest GWAS meta-analysis in ADHD available (19,099 cases and 34,194 controls) [4]. The SNP-wise mean model was used as the statistical test, considering the *p*-values for SNPs located within the transcribed region. Only the genes located in autosomal chromosomes could be investigated, as those variants within the sex chromosomes had not been analyzed in any of the GWAS meta-analyses included in the study. For multiple testing correction, we considered both the Bonferroni correction (112 genes; *p* < 4.46 × 10^−4^) and 10% FDR.

### 2.5. Gene-Based Analyses and Meta-Analysis with Comorbid Conditions

For the genes significantly associated with ADHD, we inspected which comorbid traits were present in the OMIM database. We performed a gene-based analysis, as previously described, on: anxiety [14], ASD [5], children’s aggressive behavior [15], impulsive personality traits [16], and OCD [17] (Supplementary Table S2). All GWAS summary statistics were obtained from the Psychiatric Genomics Consortium (PGC; https://www.med.unc.edu/pgc/download-results/, accessed on 10 April 2019), except for impulsive personality traits, where results from the gene-based association analyses were obtained directly from the published manuscript (see Supplementary Table S2).

For significantly associated phenotypes, we performed a gene-based meta-analysis to combine the results of ADHD and each comorbid phenotype using MAGMA 1.06. First, a gene-based analysis of each independent dataset was performed, as previously described. Then, the weighted Stouffer’s Z method was used to combine the Z-scores for each gene across cohorts, with weights set to the square root of the sample size. We only considered the results from the combined analyses when nominal associations were found both in ADHD and the comorbid phenotype separately and when their statistical significance increased by at least one order of magnitude in the combined analysis.

### 2.6. MetaXcan

We assessed whether the estimated expression of the OMIM genes associated with ADHD is altered in this disorder using MetaXcan [18] and the GWAS summary statistics of ADHD previously described [4]. Prediction models were constructed using the SNPs located within ± 1 Mb from the transcription start site (TSS) of the implicated genes, and were trained with the RNA-Seq data of 13 GTEx brain tissues [19]: amygdala, anterior cingulate cortex (BA24), caudate nucleus, cerebellar hemisphere, cerebellum, cortex, frontal cortex (BA9), hippocampus, hypothalamus, nucleus accumbens, putamen, spinal cord cervical c-1, and substantia nigra. The SNP covariance matrices were generated using the data of European individuals of the 1000 Genomes Project Phase 3 [12].

### 2.7. Effect on Subcortical Brain Volumes

We explored whether common genetic risk variants on the OMIM genes have an effect on subcortical brain volumes, using the summary statistics of a GWAS meta-analysis of seven MRI volumetric measures from the ENIGMA Consortium: amygdala, caudate nucleus, hippocampus, nucleus accumbens, pallidum, putamen and thalamus [20]. This GWAS meta-analysis consists of seven million markers inspected in 13,171 subjects of European ancestry [20]. We applied Bonferroni corrections, setting a significance threshold at *p* = 8.3 × 10^−3^, considering six areas.

## 3. Results

### 3.1. Genes in Mendelian Disorders Presenting with ADHD Symptoms

**Figure 1:**
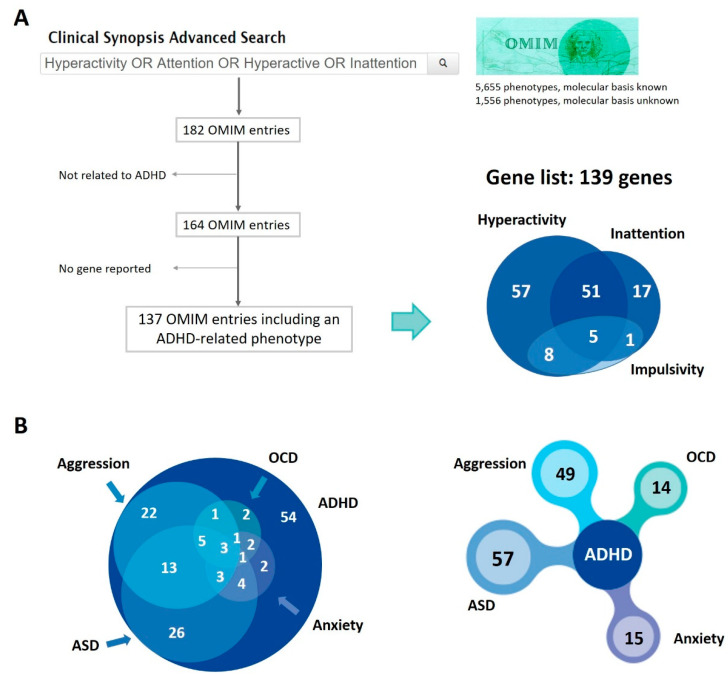
Mendelian disorders with a causal gene identified that present with ADHD symptoms. (**A**) Search in OMIM database (https://www.omim.org/; 25 February 2019) and selection, obtaining 137 disorders with a known causal gene and 139 genes related to hyperactivity and/or inattention, 14 of which also related to impulsivity symptoms. (**B**) Genes responsible for disorders that also present with other comorbid psychiatric conditions, considering the overlap between them (on the left) or only the comorbidity (on the right). ADHD: Attention-deficit hyperactivity disorder; ASD: Autism spectrum disorder; OCD: Obsessive-compulsive disorder. From the 139 genes described in the figure, 112 were located in autosomal chromosomes.

We inspected the OMIM database to search for genes involved in Mendelian disorders that present with ADHD symptoms (hyperactivity and inattention). As a result, we obtained a list of 139 genes responsible for 137 OMIM phenotypes containing at least one ADHD trait; there were 65 genes related to hyperactivity, 18 to inattention, and 56 to both symptoms (Figure 1, Supplementary Table S1). From those 139 genes, 14 were also related to impulsivity symptoms. This gene list (referred to as the OMIM gene list from now on) is enriched in genes involved in cognition, synapse organization, forebrain development, glutamatergic synapses, nicotine addiction, and metabolic processes (Supplementary Tables S3 and S4), being all consistent with the impairments, dysregulations, or comorbidities observed in ADHD patients.

We further annotated whether these 137 disorders also presented with other psychiatric or neurological comorbidities, frequently observed in ADHD patients, such as autism or aggressive behaviour. From the list of 139 genes known to cause these disorders, 57 genes were related to disorders that also present with symptoms of ASD, 49 with aggression, 15 with anxiety, and 14 with OCD (Figure 1, Supplementary Table S1). Other psychiatric symptoms were also present in some of these 137 Mendelian disorders, but less frequently (Supplementary Table S1). Neurological phenotypes, such as intellectual disability, seizures, or sleep disorders, were quite frequent among them (Supplementary Table S1).

### 3.2. Rare Variants in the OMIM Gene List and ADHD Symptomatology in Patients

We tested the possible contribution of rare variants to hyperactivity/impulsivity symptom dimensions and to inattention symptoms in a clinical sample of 668 adults with persistent ADHD. From the original list of 139 OMIM genes, we were able to extract the set of rare variants from 111 autosomal genes, with 101 of them presenting at least one high-impact rare variant (Supplementary Table S5).

We analyzed the association of these genes in a gene-based analysis for inattention and hyperactivity/impulsivity symptom dimensions. After accounting for multiple testing, we observed that three genes, harboring rare high-impact variants (*KIF11*, *WAC*, and *CRBN*), were significantly associated with inattention symptoms in this sample, with inattention being present in the Mendelian disorders they are responsible for (Table 1). On the other hand, no genes carrying rare high-impact variants were found to be associated with the hyperactivity/impulsivity symptom dimension.

Subsequently, we used a permutation procedure to assess if the burden of high-impact variants identified in the OMIM gene list was higher than expected due to chance. We identified 667 high-impact variants in 103 OMIM-genes. We observed a trend close to statistical significance (*p* = 0.057) for the enrichment of rare variants in the permutation-based analysis.

### 3.3. Common Variants in the OMIM Gene List and ADHD

We analyzed the contribution of common genetic variants in the OMIM gene list to ADHD, using a gene-based approach on the largest GWAS meta-analysis of the disorder, which included 19,099 cases and 34,194 controls [4]. We tested a total of 112 genes (in autosomes), and found six OMIM genes significantly associated with ADHD (based on 10% FDR correction). From these, five genes were related to hyperactivity (*MANBA*, *UQCC2*, *HIVEP2*, *FOPX1*, and *KANSL1*) and one with a short attention span (*AUH*) (Table 2). *HIVEP2* and *KANSL1* were also related to impulsivity. The *MANBA* gene remained associated with ADHD even after a stringent Bonferroni correction and also surpassed the gene-wide significance threshold (*p* = 5.99 × 10^−8^).

We explored whether the six genes associated with ADHD were predicted to be differentially expressed in cases versus controls in different brain areas, using MetaXcan (Supplementary Table S6). We observed that *MANBA* is differentially expressed in the cerebellum (Z-score = 3.96; *p* = 7.38 × 10^−5^) and in the cerebellar hemisphere (Z-score = 5.23; *p* = 1.63 × 10^−7^). It is important to note, however, that the information for most gene–tissue combinations was not available. We also inspected if common genetic risk variants in these six genes were associated with changes in subcortical brain volumes, using the summary statistics of a GWAS meta-analysis of seven MRI volumetric measures from the ENIGMA Consortium [20]. We found that genetic variants in *HIVEP2* were associated with changes in the nucleus accumbens (*p* = 4.87 × 10^−3^), and *AUH* and *MANBA* in the pallidum (*p* = 2.37 × 10^−3^ and 8.12 × 10^−3^, respectively). We also identified nominal associations for the *MANBA* gene with subcortical volumes of the hippocampus (*p* = 0.032) and the thalamus (*p* = 0.045).

### 3.4. Contribution to Comorbidities of the Genes Associated with ADHD

Following the previously identified ADHD-associated genes, we observed that all the Mendelian disorders with hyperactivity symptoms also presented other psychiatric comorbid traits such as ASD, aggression, anxiety, impulsivity, or OCD (Supplementary Table S1). We inspected those genes in GWAS of these comorbid traits and found associations for most of them, consistently with the symptomatology in those Mendelian disorders (Table 2).

For ASD we found associations with the genes *HIVEP2*, *FOXP1*, and *KANSL1* using a GWAS of 18,382 cases and 27,969 controls (Table 2 and Supplementary Table S7). Associations for *HIVEP2* and *FOXP1* were nominal, but gene-wide significant for *KANSL1* (*p* = 7.34 × 10^−7^). The gene *UQCC2* did not show association with ASD.

In the case of anxiety, we found a nominal association with the *HIVEP2* gene using a GWAS of 7016 cases and 14,745 controls (Table 2 and Supplementary Table S8). The *KANSL1* gene did not show association.

For aggression, *FOXP1* was found nominally associated in a GWAS of 18,988 individuals (Table 2 and Supplementary Table S9). Neither *MANBA* nor *UQCC2* were associated with aggression.

For OCD, no significant association with *FOXP1* was identified using a GWAS that included 1773 cases, 6122 controls and 915 trios (Table 2).

In all cases, we observed a more robust signal for these associations (at least one order of magnitude larger) when combining ADHD with the comorbid trait in a gene-based meta-analysis (Supplementary Tables S7–S9). We did not identify any association between the six genes and impulsivity or OCD.

Interestingly, *KIF11,* that was significantly associated with ADHD in the analysis of rare variants, was also nominally associated with aggression, consistently with the symptomatology in this Mendelian disorder (Supplementary Tables S1 and S9).

## 4. Discussion

In our study, we followed a new approach to identify novel risk genes for ADHD. We searched for Mendelian disorders with a known causal gene that present with hyperactivity and/or inattention among their symptomatology, and identified 137 OMIM phenotypes and 139 genes related to them. They are enriched in genes involved in neurological processes, brain function, and development. Nine of those genes were highlighted in our study, three of which showing association with rare variants related to the severity of ADHD symptoms (*KIF11*, *WAC*, and *CRBN*) and six carrying common genetic variants that are associated with ADHD (*MANBA*, *UQCC2*, *HIVEP2*, *FOPX1*, *KANSL1*, and *AUH*). Furthermore, for *HIVEP2*, *FOXP1,* and *KANSL1*, which are causal genes for rare disorders presenting with psychiatric conditions that are frequently comorbid with ADHD, we also found associations with these comorbidities.

A total of 137 rare disorders with an ADHD-related phenotype were identified, representing 2.2% of the 6138 OMIM entries with a known molecular basis. A similar number (95 disorders) was found in a previous study investigating symptomatology featuring aggressive behavior in rare disorders [21]. Among the 139 causal genes for the rare disorders with an ADHD-related phenotype, we found an enrichment of genes involved in cognition, synapse organization, forebrain development, glutamatergic synapse, nicotine addiction, and metabolic processes. All these functions and pathways are related to this complex neurodevelopmental disorder. ADHD is characterized by deficits in multiple cognitive domains [1], and patients have a lower surface area of the prefrontal cortex [22] and a lower concentration of glutamate and glutamine in the basal ganglia [23]. Although these regions have been extensively studied in ADHD, these are not the only brain alterations related to the disorder, and other regions and neurotransmitter systems are also involved in its development. Genes implicated in synapse organization and development and glutamatergic transmission have been associated with ADHD [24,25,26,27,28]. Furthermore, nicotine addiction is more frequent in ADHD patients (42%) than in controls (26%), and the genetic correlation between both phenotypes is high (rg = 0.53, *p* = 1.85 × 10^−13^) [29]. In rodents, ADHD-like behavioral symptoms co-occur with metabolic hypoactivity of the prefrontal, mesolimbic, and subcortical brain areas [30]. The functions and pathways identified in the OMIM gene list reveal molecular mechanisms important for ADHD. Similarly, information retrieved from genes containing causal evidence for Mendelian disorders related to aggression was useful to elucidate molecular pathways and to identify genes that are relevant for aggression, when combined with genetic information obtained from GWAS or animal models [31].

Remarkably, one of the genes included in the OMIM gene list, *TBC1D2*, was pointed to as a risk gene in a previous study in three families with multiple ADHD-affected members, with an apparent dominant inheritance pattern [8]. This gene is responsible for pontocerebellar hypoplasia type 11. Patients with this disorder present with behavioral abnormalities, including attention-deficit hyperactivity, autistic features, and stereotyped behavior (OMIM #617695).

The analysis of rare variants in the OMIM gene list pinpointed three genes, *KIF11*, *WAC*, and *CRBN*, which carry high-impact genetic variants associated with inattention severity. All disorders related to these genes include ADHD among their clinical manifestations: *KIF11* is responsible for a disorder with microcephaly and lymphedema (OMIM #152950), *WAC* for Desanto-Shinawi syndrome (#616708), and *CRBN* is responsible for a recessive intellectual disability syndrome (#616708). The gene *KIF11* encodes for a kinesin, *CRBN*, which is a protein involved in ubiquitination, and *WAC*, a molecular adaptor involved in ubiquitination and several biological processes such as the cell cycle and autophagy.

Analysis of common variants highlighted six genes associated with ADHD, five of which (*MANBA*, *UQCC2*, *HIVEP2*, *FOPX1*, and *KANSL1*) were related to rare disorders presenting with hyperactivity, and one (*AUH*) with a short attention span. *MANBA* is responsible for β-mannosidosis (#248510), *UQCC2* for Mitochondrial complex III deficiency (#615824), *KANSL1* for Kooln-de Vries Syndrome (#610443), *AUH* for 3-methylglutaconic aciduria type I (#250950), and both *HIVEP2* and *FOXP1* are related to syndromes with intellectual disability (#616977 and #613670, respectively).

*MANBA*, encoding for β-mannosidase, has been found to be associated with ADHD at a gene-wide significant level in our study. The encoded protein is a lysosomal enzyme that has been especially related to kidney disease but also to psychiatric disorders. Previous studies identified *MANBA* as a gene that is associated with schizophrenia and nicotine dependence [32], as well as antipsychotic response [33].

*HIVEP2*, *FOXP1*, and *KANSL1* have been found to be associated with ADHD and also were nominally associated with ASD in our study. *HIVEP2* and *FOXP1* code for transcription factors and both have previously been related to autism [34,35,36]. *HIVEP2* has also been linked to schizophrenia and substance use disorders [37,38,39], and knockout mice for this gene show hyperactivity, anxiety, and schizophrenia-related behaviors [40,41], which is in line with the associations we found between ADHD and anxiety. *FOXP1* was also found to be associated with aggression in our study, but, to our knowledge, it has not been previously related to aggressive behavior. One of the *loci* associated with ADHD in the largest GWAS meta-analysis pointed to *FOXP2*, another member of the same family [4]. *FOXP2* forms homo- and/or heterodimers with *FOXP1* and *FOXP4* [42,43], and regulates neuron maturation and locomotor activity [44]. Thus, these three genes seem to have a pleiotropic role in ADHD and its related comorbidities.

Several limitations of this study should be taken into account. On one hand, the sample size for the rare variant analysis is limited, and this could have prevented us from identifying associations of other genes with the dimension of the severity of ADHD symptoms. On the other hand, the statistical power of the GWAS meta-analysis, used for identifying common variants, is still limited, especially for OCD and impulsive personality traits, in which we could not identify any gene associated, potentially due to the limited sample size of these studies. Also, common variants could not be investigated separately in the different symptom domains of ADHD. Causal genes for Mendelian disorders presenting only with impulsivity, and not in combination with hyperactivity, were not investigated, which could have hindered our ability to identify additional relevant genes for ADHD. Finally, rare and common variants within genes located on non-autosomal chromosomes could not be assessed in this study. Therefore, further studies would be of help to confirm the role of these OMIM genes in ADHD.

## 5. Conclusions

Overall, our results delineate an alternative way to identify candidate genes for complex disorders through exploring the shared phenotypic space with Mendelian diseases. We have seen that genes related to hyperactivity and/or inattention symptoms in Mendelian rare disorders could be relevant in the common and complex forms of ADHD, and have identified mechanisms that are potentially involved in neurodevelopment and brain function. Following this strategy, we have identified nine novel candidate genes for ADHD, three of them being of special interest for their potential pleiotropic role in several ADHD comorbidities.

## Figures and Tables

**Table 1:** Genes associated with the inattention symptom dimension in the rare variants analysis from the OMIM gene list (autosomal).

Gene Symbol	OMIM	ADHD Phenotype in Mendelian Disorder	Genomic Coordinates (hg38)	Number of Rare Variants Identified	*p*-Value	Adjusted *p*-Value
*KIF11*	#152950	Hyperactivity/inattention	10:92593068-92655395	1 (10:92613567–A)	4.81 × 10^−^^6^	2.43 × 10^−^^4^
*CRBN*	#607417	Hyperactivity/inattention	3:3149633-3179717	3 (3:3150954–G; 3:3174156–T; rs201449042)	3.03 × 10^−^^6^	2.43 × 10^−^^4^
*WAC*	#616708	Hyperactivity/inattention	10:28532588-28623112	1 (rs201855730)	1.03 × 10^−^^4^	3.45 × 10^−^^3^

**Table 2:** Significant results from the GWAS gene-based association studies on the OMIM gene list (autosomal).

Gene Symbol	Mendelian Disorder	ADHD Phenotype in Mendelian Disorder	Comorbid Psychiatric Phenotypes in Mendelian Disorder	ADHD Gene-Based *p*-Value	Adjusted *p*-Value *	Significant Gene-Based Association of Comorbidities
*MANBA*	#248510	Hyperactivity	Aggression	**5.99 × 10^−8^**	6.65 × 10^−6^	
*UQCC2*	#615824	Hyperactivity	ASD/Aggression	5.88 × 10^−4^	0.0326	
*HIVEP2*	#616977	Hyperactivity/impulsivity	ASD/Anxiety	1.10 × 10^−3^	0.0407	ASD/Anxiety
*FOXP1*	#613670	Hyperactivity	ASD/Aggression/OCD	2.43 × 10^−3^	0.0674	ASD/Aggression
*KANSL1*	#610443	Hyperactivity/impulsivity	ASD/Anxiety	3.56 × 10^−3^	0.0790	ASD
*AUH*	#250950	Short attention span	-	4.63 × 10^−3^	0.0857	

ADHD: Attention deficit/hyperactivity disorder; ASD: Autism spectrum disorders. OCD: Obsessive-compulsive disorder. In bold, overcoming Bonferroni correction. * *p*-value adjusted by False Discovery Rate (FDR).

## Supplementary Materials

The following are available online at https://www.mdpi.com/article/10.3390/genes13010093/s1, Table S1: Complete description of OMIM entries used in the study, Table S2: Summary statistics used to inspect common variants in OMIM genes, Table S3: Enrichment of genes in KEGG pathways in the OMIM gene list, Table S4: Enrichment of genes in biological processes (GO) in the OMIM gene list, Rare variants in ADHD individuals in the OMIM gene list, Table S5: Rare variants in ADHD individuals in the OMIM gene list, Table S6: Results from the MetaXcan analyses. Table S7: Results from the gene-based analysis of ADHD, ASD and the meta-analysis of both traits. Table S8: Results from the gene-based analysis of ADHD, anxiety and the meta-analysis of both traits. Table S9: Results from the gene-based analysis of ADHD, aggression and the meta-analysis of both traits.
